# Bio-Inspired Design of Bi/Tridirectionally Anisotropic Sliding Superhydrophobic Titanium Alloy Surfaces

**DOI:** 10.3390/nano10112140

**Published:** 2020-10-27

**Authors:** Jinkai Xu, Yonggang Hou, Zhongxu Lian, Zhanjiang Yu, Zuobin Wang, Huadong Yu

**Affiliations:** 1 Ministry of Education Key Laboratory for Cross-Scale Micro and Nano Manufacturing, Changchun University of Science and Technology, Changchun 130022, China; houyonggang12@163.com (Y.H.); 13514310244@163.com (Z.Y.); wangz@cust.edu.cn (Z.W.); yuhuadong@cust.edu.cn (H.Y.); 2 International Research Centre for Nano Handling and Manufacturing of China, Changchun University of Science and Technology, Changchun 130022, China

**Keywords:** three-level structure, titanium alloy, superhydrophobic, anisotropic sliding

## Abstract

Many biological surfaces with the multi-scale microstructure show obvious anisotropic wetting characteristics, which have many potential applications in microfluidic systems, biomedicine, and biological excitation systems. However, it is still a challenge to accurately prepare a metal microstructured surface with multidirectional anisotropy using a simple but effective method. In this paper, inspired by the microstructures of rice leaves and butterfly wings, wire electrical discharge machining was used to build dual-level (submillimeter/micrometer) periodic groove structures on the surface of titanium alloy, and then a nanometer structure was obtained after alkali-hydrothermal reaction, forming a three-level (submillimeter/micrometer/nanometer) structure. The surface shows the obvious difference of bidirectional superhydrophobic and tridirectional anisotropic sliding after modification, and the special wettability is easily adjusted by changing the spacing and angle of the inclined groove. In addition, the results indicate that the ability of water droplets to spread along parallel and perpendicular directions on the submillimeter groove structure and the different resistances generated by the inclined groove surface are the main reasons for the multi-anisotropic wettability. The research gives insights into the potential applications of metal materials with multidirectional anisotropic wetting properties.

## 1. Introduction

Many biological surfaces in nature exhibit directional wettability, which comes from the fact that nature provides a variety of submillimeter/micron/nanocomposite multi-scale microstructural surfaces, and the different arrangements and combinations of multi-scale structures directly affect the motion direction of water droplets [1,2,3,4,5]. Water droplets, for example, show bidirectional anisotropic sliding characteristics on rice leaves because the large-scale wavy structure and the surface with micro/nanostructure on the rice leaves forms a periodic one-dimensional arrangement, and therefore the water droplets first slide longitudinally along with the rice leaf rather than perpendicularly. Thus, this anisotropic sliding ability makes the water droplets move along the vein and finally reach the root, providing more opportunities for rice to survive in arid environments [6,7,8]; the wings of a butterfly are covered with a micro-scale structure on which the single ridge nanostripes are evenly distributed. On these nanostripes, multi-layer lamella with different lengthens are stacked stepwise, and the nanotips extend top of the nanostripes, which are tilted slightly upward. This combined micro/nanoscale structure generates periodic distribution along the wing, making water droplets easily slide off the wing along the radial outward direction but be pinned in the opposite direction. Therefore, even on rainy days, a butterfly can keep its wings dry and continue to fly [9,10]; another example is observed in beetles’ back structure. Beetles living in the desert use the combination of protrusions and depressions of the hydrophobic and hydrophilic areas on the back to capture water they need from the humid air [1,11]; this anisotropic wettability also can be found on the surfaces of some other organisms, such as the spider silk [2], legs of water strider [5], pitcher plants [4], cactus spine, and other organisms [3].

Controlling the anisotropic wettability of the surface is of great significance for basic research. Liquids can be driven along the preferred direction according to the designed structure, which has a broad application prospect in many engineering fields, including self-cleaning, water collection, anti-icing, microfluidic transmission, catalysis, and drag reduction [12,13,14,15,16,17,18,19]. In particular, it provides reliable guidance in the design and construction of microfluidic systems and directional fluid control devices [20,21,22,23,24]. To date, many researchers have used various manufacturing methods to replicate and imitate the complex structures of biological surfaces to obtain controllable wetted surfaces mainly including duplication from a natural template [25,26], layer-by-layer assembly [8,27], lithography [28,29], laser microfabrication [30,31,32], electrochemical etching/deposition [33], machining [34,35], wire electrical discharge machining [36,37,38], and other manufacturing methods. For example, Fang et al., inspired by rice leaves and butterfly wings, designed a biologic tri-anisotropic sliding superhydrophobic surface, constructed a microgroove array structure by selective femtosecond laser ablation of polydimethylsiloxane naphthene surface, and introduced a ladder structure into the microgroove to achieve tridirectionally anisotropic wetting [31]. Zhou et al. prepared a periodic array pattern composed of asymmetric multidirectional stair elements in aluminum alloy plate using selective laser texturing method and realized the superhydrophobic surface with four-way anisotropic sliding behavior for the first time, which provided a new understanding of anisotropic wettability [39]. In recent years, bioinspired superhydrophobic and multidirectional anisotropic wetting surfaces have become a research hotspot. So far, artificial surfaces with bidirectionally or multidirectional anisotropic wettability have been reported. However, it is very difficult to prepare an anisotropic wetting surface with a multi-level combination of supernatural bionic structures that is easy to control accurately [31,32,39]. Therefore, in this paper, wire electrical discharge machining (WEDM) is used to prepare a periodic inclined groove structure on the titanium alloy (Ti-6Al-4V) surface. Thanks to its high flexibility and controllability, this method not only can fabricate accurately a large-period heterostructure with a complex shape, but also produce a convex microstructure similar to that of rice leaves on the metal surface [40,41,42], and is considered one of the most competitive processing methods in generating large-scale superhydrophobic and anisotropic wetting surfaces.

Inspired by rice leaves and butterfly wings, this paper uses WEDM to prepare a two-level combination structure on the surface of Ti-6Al-4V with submillimeter inclined grooves and micron-level pits and bulges, and then a tertiary structure with nanoscale is finally obtained after alkali-hydrothermal (AH) reaction. In addition, systematically, studies have been conducted on the surface hydrophobicity and anisotropic sliding. As the asymmetric submillimeter level inclined groove structure is the key factor to form the surface hetero-wetting, while the micro/nano structure is an effective way to reduce the adhesion of the solid surface, in this work the degree of asymmetry of the groove structure is properly adjusted by designing the spacing of the inclined groove and the size of the inclination angle. As a result, a surface with bidirectional hydrophobicity and tridirectionally anisotropic sliding superhydrophobicity has been fabricated for the first time through WEDM on the Ti-6Al-4V surface. This work is expected to help improve the understanding of anisotropic wettability and provide new insights into the preparation of functional metal surfaces and the further development of microfluidic systems.

## 2. Materials and Methods

### 2.1. Materials

The Ti-6Al-4V workpiece was purchased from Changchun Heng Feng Metal Materials Co., Ltd. (Changchun, China); the chemical composition of the Ti-6Al-4V alloy is shown in Table 1; and the tool electrode, a high-precision galvanized electrode wire (AC CUT A900, Φ100 µm), was manufactured by GF company (Kinzenbach, Germany). The methanol (CH_3_OH), acetone (C_3_H_6_O), alcohol (C_2_H_6_O), and sodium hydroxide (NaOH) used in the experiment were produced by Beijing Chemical Works (Beijing, China). The deionized water used in the AH reaction was supplied by Jiangsu Xizhimeng Trading Co., Ltd. (Suqian, China). 1H,1H,2H,2H-perfluorodecyltri-chlorosilane modifier (C_13_H_13_F_17_O_3_Si) was produced by Shanghai Macklin Biochemical Co., Ltd. (Shanghai, China).

### 2.2. Preparation of Three-Level Combination Structure

**Figure 1:**
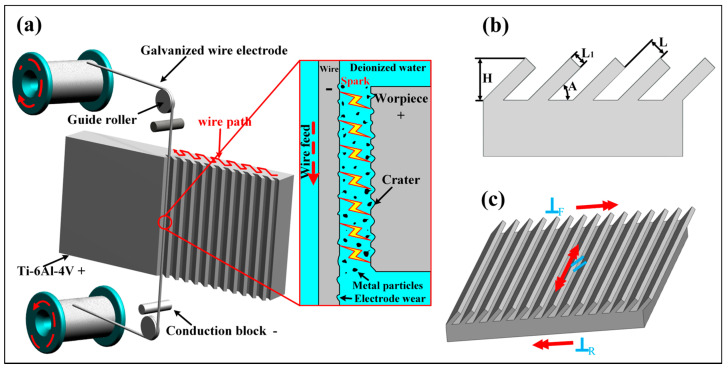
(**a**) Schematic diagram of fabrication of periodic inclined groove structure by wire electrical discharge machining (WEDM). (**b**) Side view of periodically inclined groove structure. (**c**) 3D schematic diagram of periodic inclined groove structure, three directions are defined in “//”, “⊥_F_”, “⊥_R_”.

In this research, a WEDM machine (CUT-2000X, GF company, Losone, Switzerland) was used to prepare the groove structure with periodic inclination. In this process, the electrode wire is connected to the cathode, the Ti-6Al-4V workpiece is connected to the anode, and deionized water is used as the working medium for electrical discharge machining. The WEDM machining process is shown in Figure 1a. When pulse voltage is applied to the workpiece and the electrode wire, the power supply punctures the working medium and creates a discharge that generates thousands of sparks between the wire electrode and the workpiece. Thus, thermal expansion occurs rapidly on the discharged part of the workpiece and leads to explosion which takes away melted materials, so that the workpiece material is removed. As a result, the surface exhibits a series of continuous pits and bulges as the remained materials solidify on the workpiece surface [43]. The servo system adjusts the feeding path of the electrode wire according to the shape of the programming pattern and finally processes a dual-level structure surface with a combination of submillimeter-level grooves and micrometer-level pits and bulges. In the experiment, Ti-6Al-4V with the dimension of 50 mm length × 10 mm width × 10 mm thickness. The process parameters are adjusted as follows. The discharge current is 9 A, the discharge power is 45 W, and the electrode wire speed is 130 mm/s. The structure is shown in Figure 1b, where *L* represents the width of the groove, L_1_ refers to the interval of the processing area, H is the height of the parallel groove structure, and *A* is the inclination angle of the groove and the parallel plane. The anisotropy of the groove structure surface was obtained by varying *L* and *A* (*L* = 200, 250, 300, 350 μm; *A* = 90°, 75°, 60°, 45°, 30°), and L_1_ and H remain unchanged (L_1_ is 200 µm, H is 600 µm). The WEDM processed samples were ultrasonically cleaned in acetone, absolute ethanol and deionized water, respectively, and then dried in the air.

Next, the nanostructure is fabricated on the dual-level structure surface by AH reaction. In this regard, the dual-level structured samples were immersed into a polytetrafluoroethylene container with NaOH aqueous solution (30 mL of 10 mol/L). The container was properly sealed and put in an autoclave to heat at 180 °C for 24 h. After that, the samples were taken out and rinsed repeatedly with deionized water to flush away residual NaOH, and then the samples were put in a drying oven to dry at 100 °C for 1 h, the nanostructure embedded on the surface of dual-level structures was obtained, the construction of three-level (submillimeter, micron, and nanometer microstructures) combined microstructures were completed. Finally, the prepared sample with three-level structure was immersed into the fluorosilane methanol solution for 2 h to reduce the surface energy of the sample and obtain excellent hydrophobic properties. Three directions are defined in Figure 1c: along the parallel direction of the inclined groove (//), forward the direction perpendicular to the inclined groove (⊥_F_), and reverse the direction perpendicular to the inclined groove (⊥_R_).

### 2.3. Sample Characterization

A scanning electron microscope (SEM; Quanta 250, FEI, Hillsboro, OR, USA) was used to obtain the details of the morphology of the tertiary composite microstructure on the Ti-6Al-4V surface. The surface chemical composition was analyzed using X-ray energy dispersive spectroscopy (EDS; INCAEnergy (Jena, Germany)). The static contact angles, sliding angles and water droplets sliding process were measured using an optical contact angle measuring instrument (Kruss, DSA100, Hamburg, Germany). In each measurement, 7 μL of deionized water was used to measure three different locations on the surface of each sample at room temperature, and the average value was taken as the final test result. During the measurement process, the solid–liquid contact area in the water drop profile picture taken at each position is enlarged to the same multiple, and the actual solid–liquid contact line position is marked to obtain the contact angle of the water droplet. Measurements were recorded five times to average to ensure the correctness of the results.

## 3. Results and Discussion

### 3.1. Relationship between Microstructure and Surface Wettability

**Figure 2:**
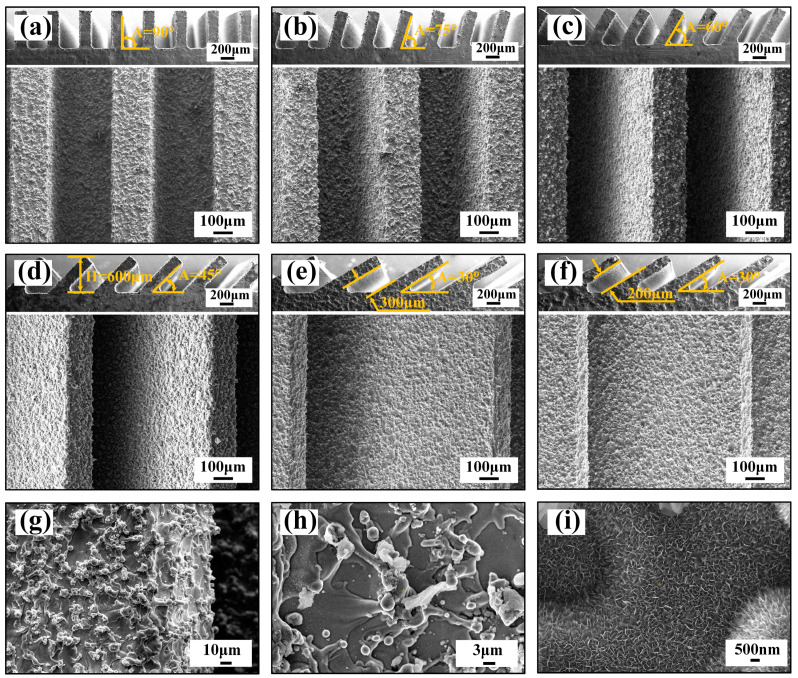
SEM images of different shapes of titanium alloy surface: (**a**–**e**) *L* = 300 μm, *A* = 90°, 75°, 60°, 50°, and 30° groove structure surface; (**f**) *L* = 200 μm, *A* = 30° groove structure surface; (**g**) micron structure formed by WEDM processing; (**h**,**i**) nanostructure formed by AH reaction.

A three-level combined bionic groove structure was prepared by using WEDM combined with AH reaction, and the surface morphology of the structure is shown in Figure 2. Figure 2a–e is the SEM photographs of the WEDM processed Ti-6Al-4V surface with different inclination angles (*L* is 300 μm, and *A* is 90°, 75°, 60°, 45°, and 30°), and Figure 2f is the image of grooves with an *A* of 30° and *L* of 200 μm. Thus, the precise construction of periodic groove structure with different inclinations and spacing values has been realized by WEDM. By further amplifying the WEDM processed surface, as shown in Figure 2g, a 10–20 μm sized tiny crater was observed on the groove surface due to local melting and vaporization of Ti-6Al-4V matrix resulting from discharge during the WEDM process while a lot of random-distributed mastoids were found around the crater structure, displaying micron-grade pits and bumps that fairly uniform in size on the processed surface. As shown in Figure 2g, the micron-grade concave bump structure was relatively smooth without noticeable nanostructures. The WEDM-processed samples were further processed by AH, and the resulting surface morphology is shown in Figure 2h. It can be observed that the pits and bulges on the surface of Ti-6Al-4V were covered with a layer of the fluff-like structure. After further magnifying, as shown in Figure 2i, polygonal micropores and nanoscale pore wall structures were observed on the surface. It can be concluded that by combining WEDM and AH, a three-level microstructure with a combination of submillimeter grooves, micrometer-level pits, and bumps, nano-level grids were fabricated on the Ti-6Al-4V surface. Large-scale construction of bionic dual-scale and multi-scale microstructures was realized.

**Figure 3:**
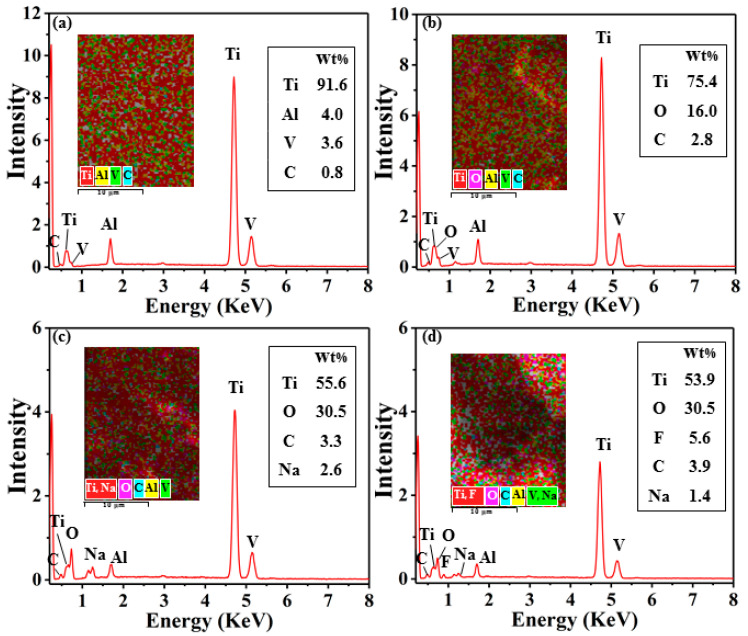
EDS pattern and chemical composition of different surface reaction methods: (**a**) polished surface; (**b**) WEDM surface; (**c**) WEDM-AH surface; (**d**) fluorosilane modified WEDM-AH surface.

EDS was used to analyze the chemical composition of the sample surfaces treated by different methods, as shown in Figure 3. As can be seen in Figure 3a, the C content on the surface increased because certain organic matters present in the air attached on the surface of the sample during polishing the Ti-6Al-4V surface. The content of C and O elements on the surface of Ti-6Al-4V increased significantly after electrical processing, as shown in Figure 3b. The presence of the O element can be explained that the material removal occurred in the aqueous medium, and the high temperature generated during discharge caused the surface to oxidize resulting in a sharp increase in O element. An increase of C element can be understood that a little organic matter in the aqueous medium decomposed at high temperature and produced a large amount of free C. As the material resolidified during the electrical machining process, free C attached to the surface, making the C content increase. On the other hand, the content of O on the surface of Ti-6Al-4V increased sharply after AH reaction, accompanied by the appearance of Na element, which increased the element O/Na ratio, as shown in Figure 3c. This is the case where the sample surface is covered with a layer of sodium titanate in a high-temperature alkaline solution environment [44]. Moreover, the content of C increased after AH reaction because a large amount of organic matter presents in the air deposited on the sample surface during the drying process. In addition, after the WEDM and AH reaction, F was observed on the surface because the samples were modified with fluorosilane. Besides, the carbon content further increased, which caused the lower surface functional groups sequentially to assemble on the surface, reducing the surface energy of the sample surface.

**Figure 4:**
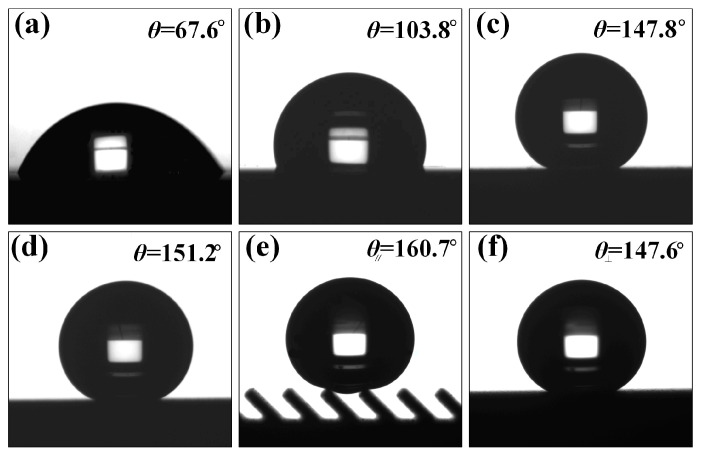
The contact angle of water with different scale structures on the surface: (**a**) smooth surface, (**b**) smooth surface after modification, (**c**) WEDM processed surface without groove structure, (**d**) WEDM-AH reacted surface without groove structure after modification, (**e**) WEDM-AH reacted inclined groove surface in the parallel direction, and (**f**) WEDM-AH reacted inclined groove surface in the perpendicular direction.

In order to study the influence of surface microstructure characteristics on the wetting property of the Ti-6Al-4V surface, the wettability of different microstructure surfaces was compared and analyzed. Figure 4 shows the contact angles of water on the Ti-6Al-4V surface with different reaction methods. It can be observed in Figure 4a that the polished Ti-6Al-4V demonstrated high surface energy and strong hydrophilicity with static contact angle of only 67.6°, as shown in Figure 4a. After the polished surface was modified with fluorosilane, the surface still failed to achieve the superhydrophobic behavior although the surface contact angle increased to 103.8. Figure 4b,c shows the measurement results of the contact angle of water droplets on the WEDM processed and fluorosilane modified surface without groove structure. The contact angle of water droplets on the surface with micron-level concave bump structure reached 147.8°, exhibiting similar superhydrophobic properties. According to the Wenzel wetting theory, if the intrinsic contact angle of the modified smooth surface is greater than 90°, the contact angle of the prepared surface will increase with an increase in surface roughness. Therefore, during the WEDM process, micro-pits and bumps structure spontaneously formed during electrical processing effectively increased the surface roughness, thereby improving the hydrophobic properties of the surface. On this basis, after AH reaction, the contact angle of water droplets on the sample surface increased to 151.2°, as shown in Figure 4d. As the construction of the composite micro-nano structure produced an increased surface roughness, the water droplets were seen to form a discrete contact with the solid surface, and hence the contact angle of the water droplets increased, forming a superhydrophobic surface. Figure 4e,f shows optical photographs of contact angles in parallel and perpendicular directions with submillimeter inclined grooves (*L* is 300 μm, *A* is 45°) and composite micro-nano structures superimposed on the surface. The appearance of the structure caused the surface of the sample to exhibit anisotropic wettability. In the direction parallel to the groove, the contact angle of the water droplet reached 160.7°, while in the direction perpendicular to the groove, the contact angle of the water droplet was 147.6°, evidencing a difference between the contact angles in both directions to reach 13.1°. From the above analysis and comparison, it can be seen that the combination of multi-level microstructures makes the Ti-6Al-4V surface exhibit good superhydrophobicity, and the submillimeter-level inclined groove structure is a key factor generating anisotropic wettability. Therefore, the three-level combined bionic structure surface prepared by the combination of WEDM and AH is one of the ideal processing methods to form an anisotropic superhydrophobic surface.

**Figure 5:**
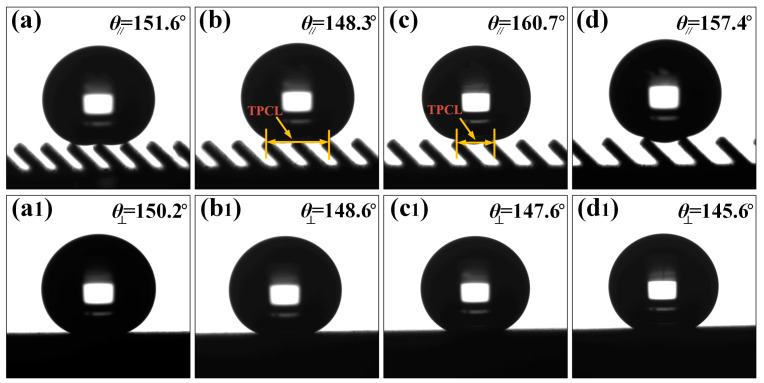
The contact angles of water droplets in the parallel and perpendicular directions at different *L* of inclined groove (*A* = 45°): the direction parallel to groove (**a**) *L* = 200 μm, (**b**) *L* = 250 μm, (**c**) *L* = 300 μm, (**d**) *L* = 350 μm; the direction perpendicular to groove (**a1**) *L* = 200 μm, (**b1**) *L* = 250 μm, (**c1**) *L* = 300 μm, (**d1**) *L* = 350 μm.

**Figure 6:**
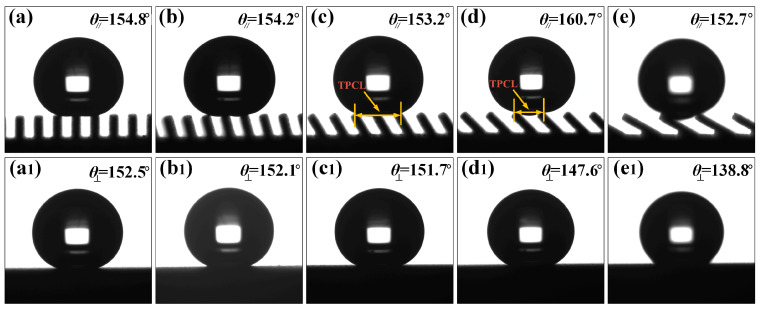
The contact angles of water droplets in the parallel and perpendicular directions at different inclination angles *A* of the groove (*L* = 300μm): the direction parallel to groove (**a**) *A* = 90°, (**b**) *A* = 75°, (**c**) *A* = 60°, (**d**) *A* = 45°, (**e**) *A* = 30°; the direction perpendicular to groove (**a1**) *A* = 90°, (**b1**) *A* = 75°, (**c1**) *A* = 60°, (**d1**) *A* = 45°, (**e1**) *A* = 30°.

To further investigate the influence of the submillimeter scale groove structure on the wettability of water droplets, the contact angle of the surface of the inclined groove structure was measured and analyzed. Figure 5 shows the static contact angles in parallel and perpendicular directions at the inclination angle *A* of the water droplet on the groove of 45° and spacing *L* of 200 μm, 250 μm, 300 μm, and 350 μm, respectively. As shown in Figure 5b, when *L* was 250 μm in the direction parallel to the grooves, the contact angle of water droplets on its surface was 148.4°, showing a similar superhydrophobic effect. If the spacing was 300 μm, the contact angle of the water droplets on its surface reached 160.7°, exhibiting the superhydrophobic property, as shown in Figure 5c. Therefore, it can be seen that groove spacing is one of the main factors affecting the value of the contact angle of the water droplet. As the contact angle of the solid surface is related to the length of the three-phase contact line (TPCL), the contact angle can be expressed as [45]

(1) $$\theta =2\arctan\frac{2h}{l}$$

where *h* is the height of the water drop and *l* is the length of the TPCL. From the formula, it can be known that when the height of the water drop is almost the same, the shorter the length of the TPCL, the greater the contact angle. It can be seen in Figure 5b,c that when *L* was 300 μm, the number of grooves in contact with water droplets was reduced to one. After measurement, the length of TPCL was about 1.27 mm when *L* was 250 μm, and the length of TPCL was about 0.91 mm when *L* was 300 μm. It can be concluded that the shorter the TPCL, the greater the contact angle. However, by comparing panels (a) and (b), and (c) and (d) in Figure 5, it can be found that when the number of water droplets supported by the groove was the same, the contact angle of the water droplet decreased as *L* increased; at the same time, as can be seen in Figure 5, the contact angle in the direction perpendicular to the groove was significantly smaller than that in the parallel direction, and the contact angle in the perpendicular direction reduced stepwise with increasing *L*. Figure 6 shows the static contact angles in the directions parallel and perpendicular to the groove with a groove *L* of 300 μm and an inclination angle *A* of 90°, 75°, 60°, 45°, and 30°, respectively. Under the same groove *L*, a decrease in *A* caused the equivalent TPCL of the parallel contact between the water droplet and the groove end surface to increase. Therefore, the contact angle of the groove in both directions has the same change rule as the groove *A* decreases and *L* increases.

**Figure 7:**
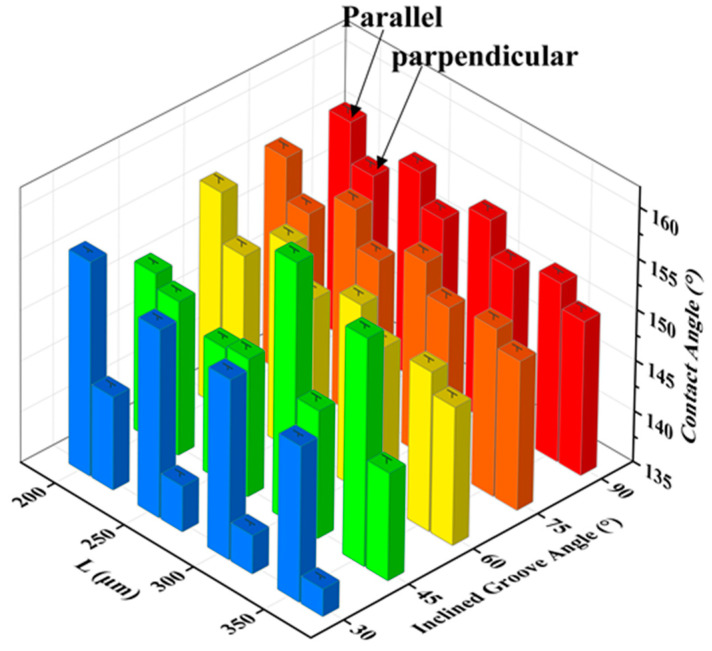
The change of contact angles in directions parallel and perpendicular to inclined groove surfaces with different inclination angles with *L*.

To further explore the relationship between the contact angles in two directions and the sub-millimeter groove structures *L* and *A*, the contact angles of water droplets on the groove surface in parallel and perpendicular directions with different structural parameters were measured. It can be seen in Figure 7 that the contact angles in the parallel direction of the micro-groove structure surface were larger than those in the perpendicular direction and the water droplets had anisotropic wetting property on the surface. The mechanism of anisotropic wetting is shown in Figure 9f. When the water droplet diffused along the direction perpendicular to the groove, it would be affected by the gap of the groove and the groove produced a peg effect to pin the three-phase contact line of the water droplet, hindering further wetting; while the water droplet spread parallel along the groove, the end surface of the groove made continuous contact with the water droplet, and under the action of the solid surface adhesion and friction resistance, it diffused smoothly in the groove until it reached equilibrium, making the length of TPCL in the parallel direction of the groove smaller than that perpendicular to the groove. As a result, the contact angle of the water droplets in the parallel direction of the groove was larger than that perpendicular to the groove, creating heterogeneous wettability. By comparing the changing trend of the contact angle of the water droplet on each inclined groove sample with groove *L*, it can be clearly seen in Figure 7 that the contact angles of the groove in both directions decreased with increasing *L* and decreasing *A* except that the contact angle in the parallel direction of the groove showed a significant change with the groove *A* of 45°. This is because an increase in *L* caused the number of water droplets immersed in the groove to increase gradually, resulting in an increased TPCL, and therefore the contact angles in both directions decreased with increasing *L*; when *L* was constant, the parallel distance of the end surface of the groove supporting the water droplets gradually increased as the inclination angle *A* of groove decreased, and thus the contact angles in perpendicular directions decreased stepwise, showing the same trend with the situation where *L* was increased. For the anomalous CAs values at 45° and 30° presented in Figure 7, the reason is that the number of grooves supporting water droplets in the parallel direction is reduced in the grooves (Figure 5b,c and Figure 6c,d), resulting in a sharp decrease in TPCL, the groove surface shows a larger contact angle. Therefore, it can be concluded that when the number of grooves supporting water droplets is the same, as *L* increases, the water droplet contact angle in the parallel direction decreases as *L* increases, as shown in Figure 7.

### 3.2. Anisotropic Sliding Performance of Water Droplets

**Figure 8:**
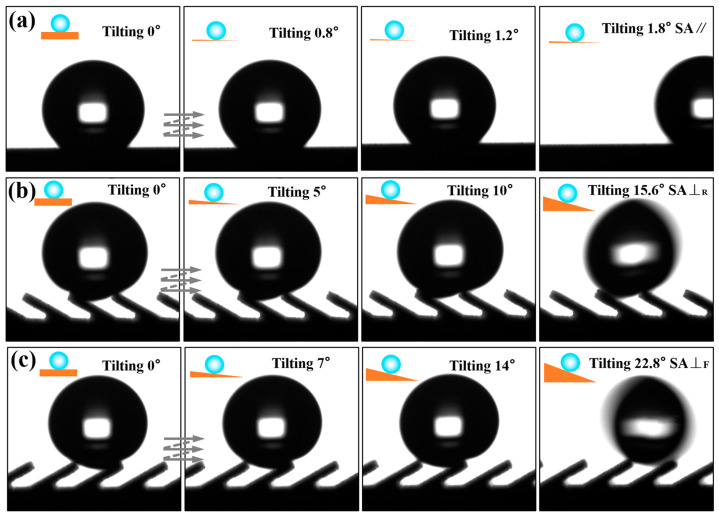
The sliding angles of water droplet in different directions on the inclined groove surface (*L* = 300 μm, *A* = 30°): (**a**) sliding along the direction parallel to the inclined groove; (**b**) reversely sliding in the direction perpendicular to the inclined groove; (**c**) forward sliding in the direction perpendicular to the inclined groove.

**Figure 9:**
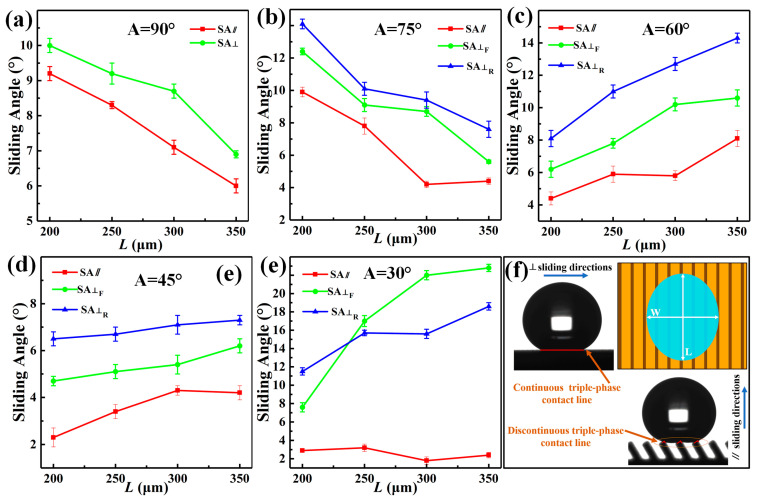
Schematic diagram of the change of the sliding direction on the groove surfaces with different inclination angles with *L* and the anisotropic wetting: the angle of the inclined groove (**a**) *A* = 90°; (**b**) *A* = 75°; (**c**) *A* = 60°; (**d**) *A* = 45°; (**e**) *A* = 30°; (**f**) Schematic diagram of anisotropic wetting.

The three-level combined and inclined groove structure prepared by the method of WEDM and AH realizes the three-dimensional anisotropic sliding of water droplets. As shown in Figure 8, 7 μL water droplets sliding on the prepared Ti-6Al-4V inclined groove structure surface (*L* = 300μm, *A* = 30°) were photographed in three directions. It is clear that the water droplet had the smallest sliding angle in the direction parallel to the inclined groove. As shown in Figure 8b, in the direction perpendicular to the inclined groove, the maximum SA⊥_F_ was 22.8° when sliding perpendicular to the inclined groove due to the influence of the asymmetric heterostructure; Figure 8c indicates that the SA⊥_R_ was 15.6°when sliding perpendicular to the inclined groove reversely. It can be seen that the Ti-6Al-4V surface of the three-level combined and inclined groove structure displayed obvious tridirectionally anisotropic sliding characteristics. In order to further explore the influence of the structural parameters of the inclined groove on the droplet sliding performance, we further measured the sliding angles of the groove surface under different structural parameters. Figure 9 illustrates the images of water droplets sliding on the groove structures with different inclination angles changing with the groove *L*. At a 90° angle, the upper end of the groove was parallel to the parallel plane. The water droplets were subject to the same resistance when sliding in both directions of the perpendicular groove, exhibiting only one kind of sliding characteristics. As can be seen in Figure 9a, the groove surface only showed the anisotropy in two directions at a 90° angle. However, when the groove was inclined, the upper end of the groove was at a corresponding angle to the parallel plane, and the surface formed an asymmetrical-shaped structure. The resistance of the water droplet was different when sliding in two directions perpendicular to the groove, showing two sliding characteristics. At the same time, the influence of different forms of solid–liquid contact lines in the perpendicular and parallel directions made the inclined groove surface exhibit tridirectionally anisotropic sliding characteristics, as shown in Figure 9b–e. When the inclination angle *A* was 75°, 60° and 45°, the sliding angles in three directions on the surface can be expressed as SA⊥_R_ > SA⊥_F_ > SA//; if *A* was 30° and *L* was greater than 200 μm, the sliding angles in three directions on the groove structure surface can be described as SA⊥_F_ > SA⊥_R_ > SA//. Due to the influence of the groove pitch *L* and the inclination angle *A* on the sliding process of water droplets, when the inclination angle *A* of the groove was 90° and 75°, respectively, the sliding angles in the directions parallel and perpendicular to the groove surface decreased with increasing *L*, as shown in Figure 9a,b, while the inclination angle *A* was 60°, 45°, and 30°, respectively, the tridirectionally anisotropic sliding angles increased as *L* increased, as shown in Figure 9c–e.

### 3.3. Mechanism of Anisotropic Sliding Behavior of Water Droplets

This section studied and analyzed the anisotropic sliding behavior of water droplets on the surface of the prepared three-level inclination groove structure so as to understand the influence of groove structure parameters on the anisotropic sliding performance of water droplets. As a matter of fact, there are several factors affecting the sliding angle when water droplets slide on a solid surface. According to the theory of Furmidge and Frenkel, the sliding angle α of the solid plane can be described as follows [7],

(2) $$mg(\sin a)=2R\gamma_{LG}(\cos\theta_{R}-\cos\theta_{A})$$

where *θ_A_* is the advancing angle, *θ_R_* is the receding angle, m is the mass of the droplet, *R* is the contact radius of the droplet and the solid surface, and *γ_LG_* is the interfacial tension between the liquid and the gas. The left part of the equation is the driving force of the water droplet on the surface under the influence of gravity, and the right part is the sliding resistance caused by the surface adhesion. It can be seen from the formula that when the water droplet quality was constant, the smaller the adhesion resistance of the water droplet, the smaller the sliding angle of the surface. The adhesion of sliding water droplets was closely related to the morphology of the surface microstructure. A decrease in *R* caused the contact area between the water droplets and the solid surface to decrease, leading to a smaller adhesion resistance of the water droplets on the surface. *R* is mainly affected by large-scale grooves. Specifically, the diffusion degree of water droplets on the surface was different with a change of *L* and *A* of the groove, resulting in varied *R*. On the other hand, for the surface with microstructures, the combination of micro- and nanostructures established discrete contact between the solid and the liquid to avoid the intrusion of external water droplets, which effectively reduced the contact area between the liquid and the solid, thereby effectively reducing the adhesion resistance of the surface [6]. The combination of the two methods will effectively reduce the adhesion resistance of the solid surface, resulting in a very low sliding angle. Therefore, a bionic groove structure with a three-level combination was prepared by a process method combining WEDM and AH reaction, so that the sliding of water droplets in the direction parallel to the groove was kept within 10°, and the minimum sliding angle reached 1.8° at the suitable submillimeter groove structure parameters.

Due to the influence of the large-scale groove structure, the water droplets spread differently along with the parallel and perpendicular directions of the groove. The diameters of the TPCL in the two directions are shown in Figure 9f. When sliding in the parallel direction, the diameter of the solid-liquid junction was short, and the adhesion resistance was relatively small; the diameter of the solid-liquid connection was long when sliding in the perpendicular direction showing a discontinuity. Not only did the water droplet experience a large adhesion resistance when sliding, but also the water droplet needed to overcome a larger energy barrier during sliding [19,20] due to the discontinuous radius of the solid–liquid connection, which can explain why the sliding angle in the direction parallel to the groove smaller than that in the perpendicular direction.

**Figure 10:**
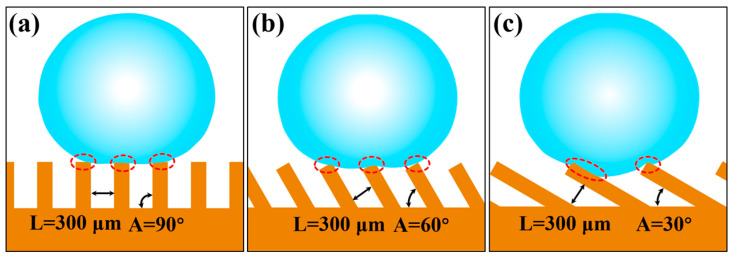
Schematic diagram of water droplets on different inclined groove (*L* = 300 μm) structures: (**a**) *A* = 90°; (**b**) *A* = 60°; (**c**) *A* = 30°.

In addition, when the groove changed from a right angle to an inclined angle, the upper-end surface of the inclined groove formed an asymmetric inclined surface. When the water droplet slid in the perpendicular direction, it presented two scenarios. The water droplet sliding forward along the direction of the inclined groove appeared as a release effect while sliding reversely along the direction of the inclined grooves exhibited a pinning effect. The sliding resistance of the water droplets under different sliding modes of action was different, demonstrating bidirectionally anisotropic sliding characteristics in the perpendicular direction [46]. Therefore, the surface of the inclined groove exhibited tridirectionally anisotropic sliding characteristics. As shown in Figure 10a, when the inclination angle *A* of inclined groove was 90°, the upper-end surface of the groove was parallel to the parallel plane, and the entire groove surface only exhibited bidirectionally anisotropic sliding property. However, as the inclination angle *A* decreased, the end face perpendicular to the groove direction produced an asymmetrical shaped structure, as shown in Figure 10b,c. The water droplets demonstrated tridirectionally anisotropic sliding characteristics on the entire inclined groove surface.

**Figure 11:**
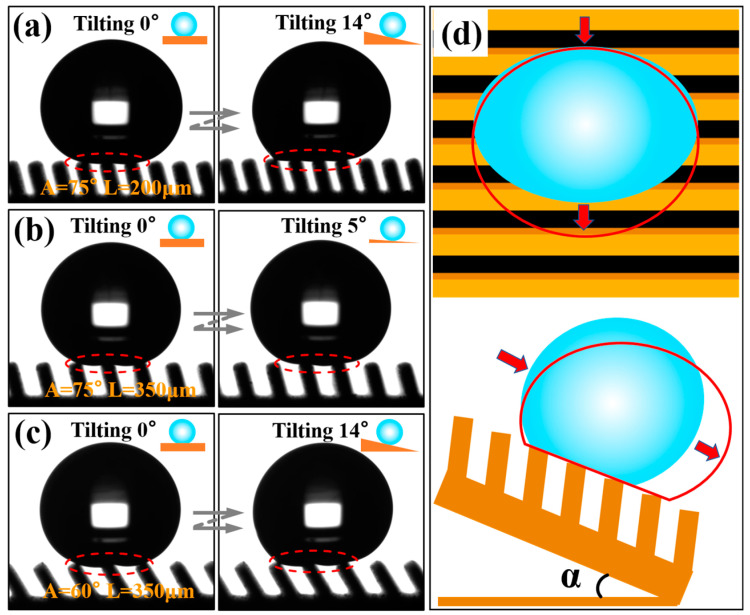
The effect of different structural parameters of the groove on the sliding angle: (**a**) *A* = 75°, *L* = 200 μm; (**b**) *A* = 75°, *L* = 350 μm; (**c**) *A* = 60°, *L* = 350 μm; (**d**) Top and side views of the evolution of TPCL during sliding of water droplet on the surface of groove *A* = 75° and *L* = 200 μm.

Anisotropic sliding was greatly affected by the submillimeter inclined groove structure size, as shown in Figure 10. When the inclination angle *A* of the groove was 75° and 60°, respectively, it could effectively avoid the intrusion of external water droplet due to the small horizontal distance between the grooves, and the water droplets were always in a non-wetting state on the inclined groove surface. Under this circumstance, when the water droplet slid forward along the direction perpendicular to the inclined groove, they were subjected to the energy barrier generated by the discontinuous TPCL and the adhesion force of the inclined end face of the groove, while water droplets sliding reversely along the inclined groove in the perpendicular direction were supported by the inclined end surface of the groove, so that the resistance of the water droplets sliding reversely along the inclined groove direction was greater than that sliding forward along the inclined groove direction, as shown in Figure 10b and Figure 11c. It can be concluded that when the angle *A* of the inclined groove was 75°, 60°, and 45°, respectively, the sliding angles in the tridirectionally appeared as SA⊥_R_>SA⊥_F_>SA//. In addition, the energy barrier generated by the discontinuous TPCL in the unwetted state and the resistance produced by the tilted end face of the groove were relatively low, the difference between the sliding angles in each direction was also very limited. However, at a 30° angle, the droplet began to be supported by one inclined groove, and the external water droplet were immersed in the groove and contact the trench wall because the horizontal distance between the inclined grooves was enlarged as the inclination angle became smaller, as shown in Figure 10c. Water droplet sliding along the direction perpendicular to the groove were subjected to greater resistance, which increased the sliding angle and broadened the difference between the sliding angles in each direction, as shown in Figure 9e. When *L* was greater than 200 μm, the sliding angles in three directions showed the change rule of SA⊥_F_>SA⊥_R_>SA//. This is because at a 30° degree, the contact area between the water droplet immersed in the groove and the two side walls of the groove was asymmetric, as shown in Figure 8b and Figure 10c. SA⊥_R_ was relatively small because the wall adhesion force worked against the gravity of the water droplet when the water droplet slid reversely in the direction perpendicular to the inclined groove. On the other hand, if the water droplet slide forward along the direction perpendicular to the inclined groove, in addition to overcoming the adhesive force on the inclined groove wall, the gravity of the water droplet accumulated directly above the inclined surface of the groove wall with increasing sliding angle, which caused greater adhesive force on the groove wall, thereby making SA⊥_F_ increase.

As shown in Figure 9a,b, when the inclination angle of the inclined groove was at 90° and 75°, the sliding angle in each direction decreased as *L* increased. By analyzing the sliding behavior of the water droplets in the dynamic video, it can be observed that if the inclination angle of the groove end surface and *L* was small, water droplets were deformed under the combined action of the adhesion force of the groove end surface and their own gravity during the sliding process. As the tilt angle of the sample increased, the number of grooves supporting water droplets increased since the deformed water droplet surface contacted the end surface of the groove, as shown in Figure 11a, which in turn led to an increased solid–liquid contact radius and increased surface adhesion force as well as increased sliding angle. The change process of TPCL during the sliding process is shown in Figure 11d. However, an increase in *L* caused the solid–liquid contact area between the water droplet and the groove surface to decrease. Moreover, the water droplet, driven by its own gravity and insufficient deformation degree, rolled off the groove surface during the sliding process, as shown in Figure 11b. When the inclination angle of the groove reduced, the inclination of the end face of the groove increased, and therefore the pinning effect became enhanced when the water droplets slide. Meanwhile, the equivalent solid-liquid contact distance in the parallel direction of a single groove increased with decreasing inclination angle and increasing *L*. Consequently, the number of water droplets immersed in the groove increased as *L* increased, as a result, the pinning effect of water droplets on the inclined end face of the groove was further increased [7,31], as shown in Figure 11c. Therefore, when the inclination angle *A* of the groove was 60°, 45°, and 30°, the sliding angles in the three directions increased with increasing *L*.

### 3.4. Tridirectionally Anisotropic Superhydrophobic Titanium Alloy Surface Water Droplet Condensation Test

**Figure 12:**
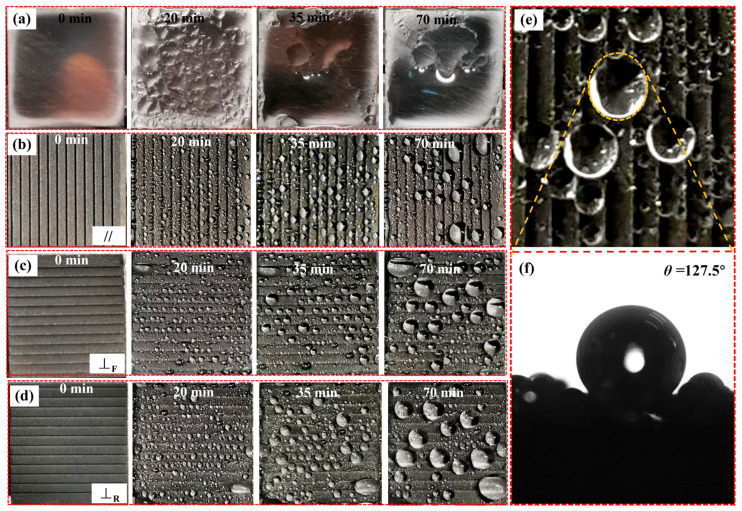
The condensation process of water droplets on different surfaces: (**a**) Smooth titanium alloy surface; (**b**–**d**) Superhydrophobic titanium alloy surface//, ⊥_F_ and⊥_R_ direction; (**e**) Condensed water droplets state on the superhydrophobic titanium alloy surface, (**f**) Condensed water droplet contact angle.

To study the effect of the designed tridirectionally anisotropic superhydrophobic titanium alloy surface on the condensation of water droplets, a water droplet condensation test was carried out. First, we used thermal grease to fix the test piece on the DSA100 refrigeration plate inclined 10°, the temperature was controlled at 1 °C, and then during the test, the indoor temperature and humidity were kept at 25 ± 2 °C, 85 ± 10%. Figure 12 shows representative images of water droplets condensing on polished titanium alloy surface and tridirectionally anisotropic superhydrophobic titanium alloy surface (*A* = 30°, *L* = 300 μm).

As shown in Figure 12a, condensed water droplets rapidly condensed into a smaller water film on the smooth surface of titanium alloy, a large water film formed as the condensation continued. However, smaller oval droplets condense on the surface of the superhydrophobic titanium alloy. This is the submillimeter groove structure that regulates the growth of the water droplets [32], inhibits the coalescence of the water droplets, and makes the water arranged along the parallel direction of the inclined grooves as shown in Figure 12b–d. The micro-nano composite structure on the surface effectively reduces the adhesion of the liquid droplets to the solid surface. When the water droplets condense to a critical size, they begin to slide and coalesce from the surface, making the water droplets gradually increase. The water droplets increase to a certain extent and begin to slide off the surface [47,48]. Due to the tridirectional anisotropy wetting in the inclined groove, the diameter of condensation droplets in the direction of the parallel groove is small. The number of droplets on the whole surface is relatively large, as shown in Figure 12b. The diameter of condensation droplets existing in the perpendicular direction is large, and the number of droplets on the whole surface is small, as shown in Figure 12d. Because the adhesion force in the parallel direction is small, the water droplets with smaller condensation can slide. The wall resistance in the perpendicular direction of the groove is relatively large; the condensed water droplets need to overcome more excellent resistance to slide. Place the refrigerating plate water horizontally. After 480 min, the sample is covered with oval droplets of different sizes, as shown in Figure 12e. The contact angle of the water droplets is 127.5°, showing hydrophobic properties, as shown in Figure 12f.

## 4. Conclusions

A periodic sloped groove structure with a submillimeter/micro/nano three-level combination was prepared on the surface of Ti-6Al-4V by the combined process of WEDM and AH. The surface of the Ti-6Al-4V exhibited obvious bidirectional superhydrophobicity and tridirectionally anisotropic sliding properties. The results show that the smaller the number of grooves and the smaller the groove *L*, the greater the parallel directions contact angle; while the contact angle in the direction perpendicular to grooves decreases with increasing *L* and decreasing *A*. When the inclined groove *A* is large, during water droplets sliding, the adhesion and resistance of the water droplets against the inclined end surface of the groove are small, and the difference in anisotropic wettability is small; as the inclination *A* of the groove decreases, the external water droplets can gradually contact the groove wall of the microgroove and the water droplets experience greater resistance when sliding, making the bidirectional hydrophobicity and tridirectionally anisotropy sliding angle shows a more significant differences. Therefore, it is a feasible solution to use WEDM and AH for processing a large number of micro-nano structures on the Ti-6Al-4V surface to reduce the adhesion of the solid-liquid contact area, and then to adjust the hydrophobicity and anisotropic sliding in different directions by considering the submillimeter inclined groove structure, which provides a simple but effective processing method for the anisotropic wetting of commercial metal materials.

## Figures and Tables

**Table 1:** Chemical compositions of the Ti-6Al-4V (wt.%).

Ti	Al	V	Fe	C	O	Other
89.7	6.1	3.9	0.15	0.02	0.12	≤0.01
